# Final results from the large sunitinib global expanded-access trial in metastatic renal cell carcinoma

**DOI:** 10.1038/bjc.2015.196

**Published:** 2015-06-18

**Authors:** M E Gore, C Szczylik, C Porta, S Bracarda, G A Bjarnason, S Oudard, S-H Lee, J Haanen, D Castellano, E Vrdoljak, P Schöffski, P Mainwaring, R E Hawkins, L Crinò, T M Kim, G Carteni, W E E Eberhardt, K Zhang, K Fly, E Matczak, M J Lechuga, S Hariharan, R Bukowski

**Affiliations:** 1 Royal Marsden Hospital NHS Trust, Fulham Road, London SW3 6JJ, UK; 2 Military Medical Institute, Department of Oncology, 128 Szaserów Street 04-141 Warsaw, Poland; 3 IRCCS San Matteo University Hospital Foundation, Piazzale C. Golgi, 19, I-27100 Pavia, Italy; 4 San Donato Hospital, Istituto Toscano Tumori (ITT), Via Pietro Nenni, 20 52100 Arezzo, Italy; 5 Sunnybrook Odette Cancer Centre, 2075 Bayview Avenue, Toronto, ON, Canada M4N 3M5; 6 Hôpital Européen Georges Pompidou, René Descartes University Paris 5, 20 Rue Leblanc, 75015 Paris, France; 7 Seoul National University Hospital, 101 Daehak-ro, Jongno-gu, Seoul 110-744, South Korea; 8 The Netherlands Cancer Institute, Plesmanlaan 121, 1066 CX Amsterdam, The Netherlands; 9 Hospital Universitario 12 de Octubre, Avenida de Córdoba, 28041 Madrid, Spain; 10 Department of Oncology, Clinical Hospital Center Split, School of Medicine, University of Split, Spinčićeva 1 21000 Split, Croatia; 11 University Hospitals Leuven, Leuven Cancer Institute, Catholic University Leuven, Herestraat 49, B-3000 Leuven, Belgium; 12 Mater Adult Hospital, Raymond Terrace, South Brisbane, QLD 4101, Australia; 13 Christie Hospital NHS Trust, Wilmslow Road, Manchester M20 4BX, UK; 14 Azienda Ospedaliera di Perugia, via Dottori, 106156 Perugia, Italy; 15 A.O.R.N. ‘A Cardarelli', Divisione di Oncologia, via A. Cardarelli, 9–80131 Naples, Italy; 16 Department of Medical Oncology, West German Cancer Center, University Hospital Essen, University Duisburg-Essen, Hufelandstrasse 55, 45147 Essen, Germany; 17 Pfizer Oncology, 10555 Science Center Drive, La Jolla, CA 92121, USA; 18 Pfizer Oncology, 558 Eastern Point Road, Groton, CT 06340, USA; 19 Pfizer Oncology, 235 East 42nd Street, New York, NY 10017, USA; 20 Pfizer Oncology, Pfizer Italia Srl, Via Lorenteggio 257, 20152 Milan, Italy; 21 Cleveland Clinic Taussig Cancer Institute, 9500 Euclid Avenue/R35, Cleveland, OH 44195, USA

**Keywords:** renal cell carcinoma, sunitinib, tyrosine kinase inhibitor, expanded-access trial, prognosis

## Abstract

**Background:**

We report final results with extended follow-up from a global, expanded-access trial that pre-regulatory approval provided sunitinib to metastatic renal cell carcinoma (mRCC) patients, ineligible for registration-directed trials.

**Methods:**

Patients ⩾18 years received oral sunitinib 50 mg per day on a 4-weeks-on–2-weeks-off schedule. Safety was assessed regularly. Tumour measurements were scheduled per local practice.

**Results:**

A total of 4543 patients received sunitinib. Median treatment duration and follow-up were 7.5 and 13.6 months. Objective response rate was 16% (95% confidence interval (CI): 15–17). Median progression-free survival (PFS) and overall survival (OS) were 9.4 months (95% CI: 8.8–10.0) and 18.7 months (95% CI: 17.5–19.5). Median PFS in subgroups of interest: aged ⩾65 years (33%), 10.1 months; Eastern Cooperative Oncology Group performance status ⩾2 (14%), 3.5 months; non-clear cell histology (12%), 6.0 months; and brain metastases (7%), 5.3 months. OS was strongly associated with the International Metastatic Renal-Cell Carcinoma Database Consortium prognostic model (*n*=4065). The most common grade 3/4 treatment-related adverse events were thrombocytopenia (10%), fatigue (9%), and asthenia, neutropenia, and hand–foot syndrome (each 7%).

**Conclusion:**

Final analysis of the sunitinib expanded-access trial provided a good opportunity to evaluate the long-term side effects of a tyrosine kinase inhibitor used worldwide in mRCC. Efficacy and safety findings were consistent with previous results.

Sunitinib is an orally administered multitargeted inhibitor of vascular endothelial growth factor receptors (VEGFRs), platelet-derived growth factor receptors, and other receptor tyrosine kinases (Abrams *et al*, 2003; Mendel *et al*, 2003; O'Farrell *et al*, 2003). The efficacy of sunitinib demonstrated in two phase II trials of patients with cytokine-refractory advanced renal cell carcinoma (RCC) led to its conditional approval (Motzer *et al*, 2006a, 2006b). In a subsequent pivotal phase III registration trial of treatment-naive patients with metastatic RCC (mRCC), sunitinib demonstrated a significant improvement in progression-free survival (PFS) compared with interferon-alfa (median PFS 11 *vs* 5 months; hazard ratio (HR): 0.42, *P*<0.001) and in objective response rate (ORR; 31% *vs* 6%, *P*<0.001; Motzer *et al*, 2007); in addition, overall survival (OS) was longer with sunitinib (median 26.4 *vs* 21.8 months; HR: 0.821, *P*=0.051; Motzer *et al*, 2009). Given the lack of active agents available in 2005 to treat advanced RCC, a global, expanded-access trial was implemented to provide sunitinib to patients in countries where its approval had not yet been granted and to those ineligible for registration-directed trials. Initial results from this expanded-access programme confirmed the activity of sunitinib in a broader ‘real-world' population (Gore *et al*, 2009).

Here we report final results with extended follow-up of the 4543 patients treated in the expanded-access trial. This extensive database was also used in an exploratory analysis to provide further external validation of the RCC prognostic model from the International Metastatic Renal-Cell Carcinoma Database Consortium (IMDC; Heng *et al*, 2009, 2013).

## Patients and Methods

### Patients

All patients were aged 18 years or older with histologically confirmed mRCC that was either treatment-naive or previously treated. Other requirements were as follows: resolution of prior treatment toxicities, adequate organ function, no major comorbidities, the potential to derive clinical benefit from sunitinib as judged by the treating physician, and ineligibility for other sunitinib studies. Any Eastern Cooperative Oncology Group (ECOG) performance status and asymptomatic brain metastases were permitted. Full details for eligibility criteria have been previously reported (Gore *et al*, 2009). All patients gave written, informed consent.

### Study design and treatment

This was an international, open-label, expanded-access trial of sunitinib (SUTENT; Pfizer Inc., New York, NY, USA) that treated patients from participating centres in 50 countries. The primary objective of the study was to provide sunitinib to patients with mRCC and no access to the drug who might benefit from treatment. Secondary objectives included assessment of efficacy and toxicity in the overall population, as well as in subgroups of interest.

The first patient was enrolled in June 2005. Accrual discontinued on a country-by-country basis according to treatment availability, with the last patient enrolled in December 2007. All patients received oral sunitinib at a starting dose of 50 mg per day for 4 weeks on treatment followed by 2 weeks off treatment in repeated 6-week cycles (schedule 4/2). Provision was made for dose reduction to 37.5 mg per day and if needed to 25 mg per day on the basis of individual tolerance. A protocol amendment in May 2006 provided investigators with the option of administering sunitinib on a continuous daily dosing schedule (usual starting dose 37.5 mg per day). Treatment continued until disease progression, unacceptable toxicity, or withdrawal of consent. The study was approved by the institutional review board or independent ethics committee at each participating centre, and was run in accordance with the International Conference on Harmonization Good Clinical Practice Guidelines.

### Study assessments

Screening evaluations included disease assessment, physical examination, biochemistry and haematology tests, and a record of concomitant medications. Monitoring safety was mandatory at regular intervals (on days 1, 14, and 28 of cycle 1, and days 1 and 28 of subsequent cycles, until a protocol amendment (May 2006) removed the day 28 assessment in cycles ⩾3) by physical examination, ECOG performance status, haematology and biochemistry tests and cardiac function (12-lead electrocardiogram), and by recording and grading all adverse events (AEs) according to the National Cancer Institute Common Terminology Criteria for Adverse Events (Cancer Therapy Evaluation Program, 2006).

No specific schedule was dictated by the protocol; however, tumour measurement was assessed by Response Evaluation Criteria in Solid Tumors (RECIST), version 1.0 (Therasse *et al*, 2000). Assessments were performed as per the local standard of care for mRCC, with data on tumour response, PFS, and OS collected when possible. Objective response rate was defined as the number of confirmed complete plus partial responses according to RECIST (Therasse *et al*, 2000). Progression-free survival was defined as the time from start of therapy to disease progression or death from any cause (whichever occurred first). Only deaths that occurred within 28 days of the last dose were counted as PFS events; however, disease progression was not restricted to the treatment period plus the 28-day follow-up period. OS was defined as the time from start of therapy to death from any cause. Patients who were not known to be dead at the time of analysis or on the data cutoff date (September 2008) were censored on the date that they were last known to be alive.

### Statistical analysis

Because of the nature of this study, there was no pre-determined sample size nor were inferential analyses preplanned or any pre-specified hypotheses tested. All patients who received at least one dose of sunitinib were included in the current analyses (modified intention-to-treat population), except for patients with non-RECIST tumour measurements (*n*=324), who were excluded from efficacy analyses based on tumour response data. Objective response rate was calculated with corresponding exact 95% two-sided confidence interval (CI) using standard methods based on the binomial distribution. The Kaplan–Meier method was used to estimate PFS and OS, with 95% CI calculated for the median.

In an exploratory analysis, patients were grouped into favourable (0 adverse factors), intermediate (1–2 adverse factors), and poor (⩾3 adverse factors) risk categories according to the IMDC model (Heng *et al*, 2009, 2013), with median OS for each group estimated by the Kaplan–Meier method and compared by the log-rank test. Adverse factors were ECOG performance status >1, time from diagnosis to study treatment <1 year (calculated as the time from original diagnosis to the date of the first sunitinib dose in the current study), haemoglobin < lower limit of normal (LLN), calcium > upper limit of normal (ULN), neutrophil count >ULN, and platelet count >ULN. In addition, a Cox multivariate analysis was used to explore the association between OS and the IMDC prognostic factors. All *P*-values were considered exploratory.

## Results

### Patients

In total, 4577 patients were enrolled into the study, including 4543 patients who received at least one dose of sunitinib and comprised the population for analysis purposes. Baseline patient characteristics are described in Table 1. The broad trial population included patient subgroups of interest, including patients age ⩾65 years (33%) and those with ECOG performance status ⩾2 (14%), non-clear cell RCC (12%), and brain metastases (7%). Overall, 26% of patients were classified as having a poor prognosis (Table 1), according to modified risk groups based on published Memorial Sloan-Kettering Cancer Center (MSKCC) criteria (Motzer *et al*, 2002, 2004).

### Treatment exposure

Patients received a median of six cycles (range: 1–57), with median treatment duration of 7.5 months (95% CI: 6.9–7.8). Median follow-up (the time from the start of therapy until the patient was censored for survival or died, whichever occurred first) was 13.6 months (range: <1–71.3 months), and was similar for those who previously had or had not received cytokine therapy. At the time of analysis, 4298 patients (95%) had discontinued treatment. Among these patients, the most common reasons for stopping therapy were lack of efficacy (39%), death (21%), AE (16%), consent withdrawn (9%), and lost to follow-up (3%). Overall, 49% of patients required a dose reduction of sunitinib. The dose was reduced to 37.5 mg per day in 34% of patients, to 25 mg per day in 15% of patients, and to 12.5 mg per day in <1% of patients. (Seventy patients (2%) were assigned to and received 37.5 mg per day on a continuous daily dosing schedule.) Dose reductions of sunitinib occurred at a higher frequency in patients who received prior cytokine treatment for advanced RCC, compared with those who had not received cytokines (51% *vs* 47%).

### Safety

Approximately 95% of patients reported treatment-related AEs of any grade, the most frequent of which were diarrhoea (47%), fatigue (40%), nausea (36%), and decreased appetite (31% Table 2). All-grade hypothyroidism was reported as a treatment-related AE in 11% of patients, and proteinuria was reported in 1% of patients overall. (Routine testing of thyroid function was not required; however, urinalysis (dipstick protein urinalysis) to monitor proteinuria was done at screening, day 1 of cycle 2, as clinically indicated, and at the end of treatment.) The most commonly reported treatment-related grade 3/4 AEs included thrombocytopenia (10%), fatigue (9%), asthenia, hand–foot syndrome, and neutropenia (each 7%), hypertension (6%), and diarrhoea (5% Table 2). In this large population, the reported overall incidence of cardiac disorders considered treatment related was 6% (grade 3/4<2%). Rates of all-grade cardiac failure or congestive cardiac failure were <1%. Twelve patients (<1%) died as a result of a treatment-related cardiac event, including cardiac failure (*n*=4), myocardial infarction (*n*=5), and cardiac arrest, cardiopulmonary failure, and myocarditis (each *n*=1).

Sixty-eight patients (1%) died from other treatment-related (non-cardiac) AEs, the most frequent of which were cerebral haemorrhage (*n*=6), death (*n*=5), hepatic and renal failure (each *n*=4), and gastrointestinal haemorrhage, general physical health deterioration, pulmonary embolism, respiratory failure, and septic shock (each *n*=3). Among the more commonly reported AEs, two patients died as a result of thrombocytopenia and asthenia (each *n*=1), as reported by the investigators.

Serum chemistry laboratory tests showed a transient increase in median alanine aminotransferase (ALT), aspartate aminotransferase (AST), alkaline phosphatase (ALP), and lactate dehydrogenase levels during cycles 1–3 that stabilised at values near or below baseline in subsequent cycles (data not shown). Clinically significant elevations in ALT or AST were each reported as all-grade treatment-related AEs in 3% of patients, with similar incidences of both and ALP reported as all-grade AEs of any cause (Supplementary Table 1).

The incidences of non-haematological, sunitinib-related AEs of any grade in subgroups of interest were comparable to (for patients aged ⩾65 years; Supplementary Table 2; and those with RCC of non-clear histology (data not shown)) or apparently lower than (for those with brain metastases (data not shown), and ECOG performance status ⩾2; Supplementary Table 3) that in the overall population. The overall incidence of non-haematological grade 3/4 AEs was broadly similar across the subgroups and when compared with the overall population, with the exception of the elderly subgroup (⩾65 years), in which the total incidence appeared slightly higher (61%) than in patients <65 years of age (51%). Otherwise, the incidences of the most commonly reported non-haematological AEs were generally similar in both age groups (Supplementary Table 2).

Of interest, the overall incidence of grade 3/4 AEs appeared to be slightly lower in patients with ECOG performance status ⩾2 (46%) than in patients with ECOG performance status 0 or 1 (56%). Otherwise, the incidences of the most commonly reported grade 3/4 AEs were generally similar, with more apparent variation in the incidences of grade 1/2 AEs according to performance status (Supplementary Table 3).

The safety profile of sunitinib appeared generally unchanged with long-term follow-up. When the final data reported here were compared with interim results reported in 2009 (Gore *et al*, 2009), the frequency and grade of common AEs were similar, except for a numerical increase in all-grade hypothyroidism (11% *vs* 6%).

### Efficacy

A total of 4219 mRCC patients were included in the analysis of tumour response, of whom 63 patients achieved a complete response (1%) and 597 patients a partial response (14%), yielding an ORR of 16% (95% CI: 15–17; Table 3). The ORR was similar in patients with and without previous cytokine treatment, and also similar in elderly patients (⩾65 years) compared with younger patients (<65 years; Table 3). Responses were reported among patients in all subpopulations of interest (Table 3). Patients at favourable risk based on the modified MSKCC prognostic criteria fared better than those at poor risk, with ORRs of 26%, 16%, and 9% in the favourable, intermediate, and poor risk groups, respectively (as originally classified before the exploratory analysis using the IMDC model). Approximately 45% of patients in the overall population had stable disease for at least 3 months, with similar rates regardless of prior cytokine treatment, among elderly patients, and in those with RCC of non-clear cell histology; patients with ECOG performance status ⩾2 or brain metastases were less likely to achieve durable stable disease (Table 3). Progressive disease or stable disease <3 months was found in 19% of patients.

**Figure 1:**
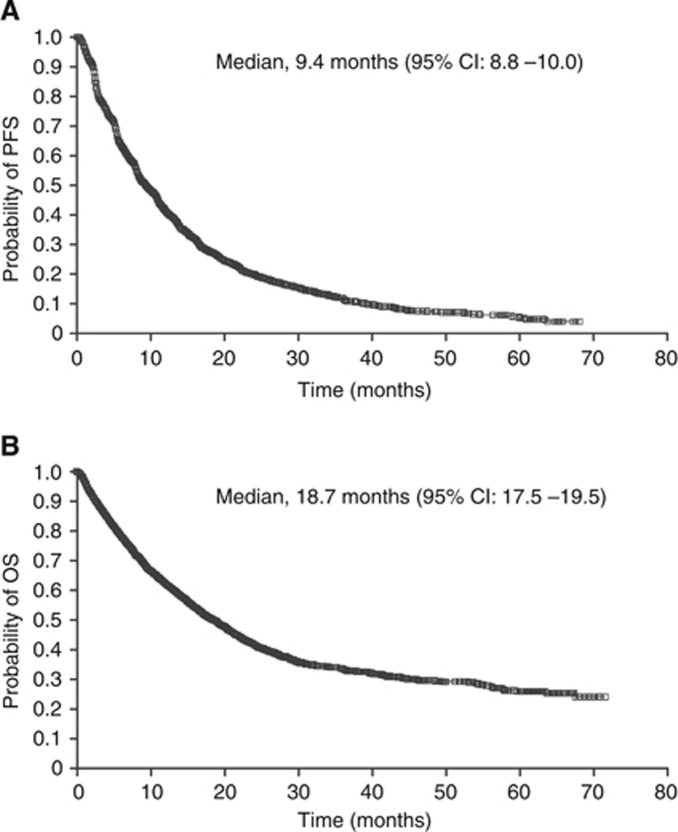
**Kaplan–Meier estimates of (A) PFS^a^ and (B) OS for the overall population.** ^**a**^In the PFS plot, 324 modified intent-to-treat patients were excluded from the evaluable population due to non-RECIST tumour assessment.

Within the limits of the lack of strictly standardised criteria for the timing and methodology of assessment of disease status, in the evaluable population, median PFS was 9.4 months (95% CI: 8.8–10.0) and median OS was 18.7 months (95% CI: 17.5–19.5; Figure 1 and Table 4). More than 20% of patients were still alive at 5 years. Median survival times were unaffected by prior cytokine status and were also similar in elderly patients compared with younger patients; however, it may have been affected by inclusion of patients with brain metastases or non-clear cell RCC (Table 4). In patients with either RCC of non-clear cell histology, brain metastases, or ECOG performance status ⩾2, both PFS and OS were substantially shorter than in the overall population (Table 4). PFS and OS also varied in patients classified according to the modified MSKCC prognostic criteria. In the favourable, intermediate, and poor risk groups, respectively, median PFS was 15.0 months (95% CI: 13.8–16.3), 10.6 months (95% CI: 9.4–11.1), and 5.4 months (95% CI: 5.1–5.7), while median OS was 56.5 months (95% CI: 41.6 to not reached), 20.0 months (95% CI: 18.4–21.3), and 9.1 months (95% CI: 8.4–9.7). The efficacy of sunitinib was maintained with long-term follow-up and was not markedly different, when final data were compared with interim results reported in 2009 (Gore *et al*, 2009; data not shown).

### IMDC prognostic model

**Figure 2:**
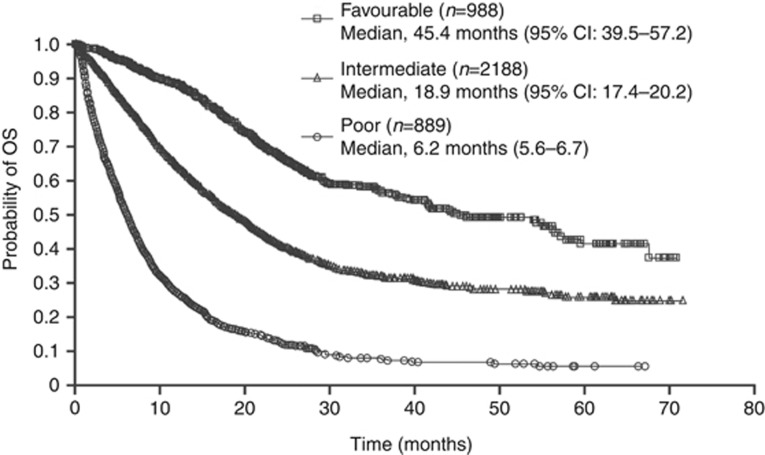
**Kaplan–Meier estimates of OS for the overall population, according to prognostic risk group based on the IMDC model (Heng *et al*, 2009, 2013). *N*=4065.** ^**a**^ ^**a**^In total, 478 patients were excluded due to missing data for one or more risk factors.

Data on the IMDC prognostic risk factors were available for a total of 4065 patients. In a multivariate Cox analysis of OS, all six factors (ECOG performance status >1, time from diagnosis to treatment <1 year, haemoglobin < LLN, calcium > ULN, neutrophil count > ULN, and platelet count > ULN) were significantly associated with reduced OS (each *P*<0.001; Table 5). OS was significantly different between patient subpopulations classified as having poor (*n*=889; median 6.2 months), intermediate (*n*=2188; median 18.9 months), and favourable risk (*n*=988; median 45.4 months), according to the IMDC model (Figure 2; for favourable *vs* intermediate or poor subgroups: HR: 0.3593, *P*<0.001; for poor *vs* favourable or intermediate subgroups: HR: 3.5153, *P*<0.001). Therefore, despite the inclusion of patients with brain metastases and non-clear cell RCC, this large sunitinib database validated external data for prognostic criteria according to the IMDC factors.

## Discussion

The final analysis of this global, expanded-access trial of sunitinib in advanced mRCC confirms the efficacy and safety of this agent in >4500 patients in a real-world setting. Although the results presented here are limited by the nature of the expanded-access study design, these findings are particularly valuable due to the large patient population. Combined with the extended duration of patient follow-up, this provides a good opportunity to evaluate sunitinib toxicity and long-term side effects. The sunitinib safety profile described here was consistent with the approved labelling for sunitinib (SUTENT (sunitinib malate) prescribing information) and provides an update to the preliminary findings from the interim report of data from this trial (Gore *et al*, 2009), with the exception of an apparent increase in the frequency of hypothyroidism (11% *vs* 6%) reported as an AE, potentially due to an increased awareness of this AE over time. The incidence of many commonly reported AEs also increased slightly, which is not unexpected given the increased treatment exposure (6 *vs* 5 cycles, respectively) and longer median follow-up (13.6 *vs* 11.6 months, respectively) in this final analysis. Low rates of all-grade, treatment-related cardiac events (6%) were observed. Efficacy outcomes were consistent with previously published findings from registration-directed phase II and III clinical trials (Motzer *et al*, 2006a, b, 2007, 2009). In those studies, the ORR was higher at 30–40%, compared with 16% here, in which tumour assessments were done as per the local standard of care for RCC. However, median PFS was 11 months in the first-line setting and 8.3 and 8.7 months in the cytokine-refractory setting, compared with 9.4 months here. OS was 26.4 months and 16.4 months in the first-line and cytokine-refractory settings, respectively, compared with our finding of 18.7 months. Notably, >20% of patients in the current study survived for 5 years or longer. In addition to subgroups of interest included in the expanded-access trial (notably patients with ECOG performance status ⩾2 or brain metastases), 68% of patients had received prior cytokine therapy and 10% had been previously treated with antiangiogenic agents. All of these factors might be expected to negatively impact on PFS and OS in the overall study population. In this context, however, it is worth emphasising that efficacy was in fact comparable in patients regardless of prior cytokine treatment.

The efficacy of sunitinib in patients with brain metastases, poor performance status, and non-clear cell RCC compares favourably with historical data for these subgroups. The safety profile of sunitinib in these three subgroups was similar to that overall, although the incidence of treatment-related AEs was consistently lower in the poor performance status and brain metastases subgroups. This may be due to shorter drug exposure; for example, patients with brain metastases received a median three cycles (range: 1–50), compared with six cycles (range: 1–57) in the overall population. Safety and efficacy in the fourth subgroup analysed, comprising elderly patients aged ⩾65 years, were broadly similar to that observed in younger patients (<65 years). This is an important finding, as this age group encompasses 64% of newly diagnosed RCC cases and accounts for >22% of deaths from this disease (Howlader *et al*, 2012). The observed activity of sunitinib in the elderly has been confirmed in an extensive retrospective analysis of data pooled from 1059 patients in six clinical trials of sunitinib as first-line or cytokine-refractory treatment for advanced RCC, which found no difference in efficacy between patients aged ⩾70 years and <70 years (Hutson *et al*, 2014). In the same analysis, although most treatment-emergent AEs occurred at similar rates in each age group, some were significantly more common in older *vs* younger patients, including fatigue, cough, peripheral oedema, anaemia, decreased appetite, and thrombocytopenia (all *P*<0.05). Despite these observations, advanced age alone is not a reason to withhold sunitinib treatment for advanced RCC, as older patients derive a similar efficacy benefit as younger ones.

Antitumour activity has also been observed in patients who received sunitinib with a primary tumour in place. In an interim analysis of data from this study, the safety profile of sunitinib was similar in patients with a prior nephrectomy and those without a prior nephrectomy (Szczylik *et al*, 2008). However, efficacy outcomes were more favourable in patients with a prior nephrectomy than in those without (e.g. median PFS, 12.0 *vs* 6.5 months, respectively; *P*=0.0021), thereby indirectly supporting the value of cytoreductive nephrectomy in the era of targeted agents, as also recently reported elsewhere (Aizer *et al*, 2014; Heng *et al*, 2014).

In an exploratory analysis, we found a strong association between OS and the IMDC prognostic factors (Heng *et al*, 2009, 2013), validating this model in >4000 patients receiving treatment in the tyrosine kinase inhibitor era. (In the original Heng *et al.* (2009), median OS was not reached in the favourable risk group; however, the 2-year OS rate was 75%, which is similar to the results in this study.) Numerous prognostic models for RCC have been developed over the past decades, in parallel with new treatment options for this disease. To our knowledge, the data reported here represent the largest contemporary patient population evaluated to date using an RCC prognostic model. That said, given the nature of our study, the characteristics of these patients, especially those in the intermediate and poor risk groups, may not be entirely equivalent to those of patients in the original Heng *et al.* (or MSKCC) model (Motzer *et al*, 2002; Heng *et al*, 2009).

The landscape for managing advanced RCC is rapidly changing, with a range of targeted agents now approved for the treatment of this disease. Despite limitations such as lack of a comparator and lack of independent review of tumour response, expanded-access trials are a practical way of offering a particular treatment to registration-directed, trial-ineligible patients and of gathering data about its activity in a real-world setting of very diverse patients. To date, the sunitinib expanded-access trial is the largest reported of its kind globally in the mRCC population. Two expanded-access trials have also been conducted with sorafenib in patients with advanced RCC, one based in the United States (*n*=2504) and one in Europe (*n*=1150; Stadler *et al*, 2010; Beck *et al*, 2011). The European trial limited patients to those in whom at least one previous line of systemic therapy had failed, or who were unable to tolerate cytokine therapy, whereas the US trial included treatment-naive patients. In each case, the safety and efficacy of sorafenib was consistent with that reported for the pivotal phase III trial (Escudier *et al*, 2007), and activity was seen in subgroups of interest similar to those included in the sunitinib expanded-access trial. Likewise, no new safety issues were identified in an international expanded-access programme of everolimus in a restricted population of patients with mRCC after failure of initial VEGFR-directed therapy (*n*=1367) (Grünwald *et al*, 2012). In this trial, median treatment duration was only 14 weeks, which may reflect the treatment setting, but which also means that safety and efficacy data gathered were limited in comparison with the sunitinib trial.

This expanded-access trial in mRCC has considerably extended our knowledge of the efficacy and safety of sunitinib in a real-world setting, and enabled over 4500 patients with wide-ranging disease states to receive sunitinib treatment. The sunitinib safety profile was consistent with prior reports, no unexpected long-term AEs were reported, and clinical benefit was seen in both treatment-naive and previously treated patients, in older as well as younger patients, and in those with traditionally poor prognosis.

## Figures and Tables

**Table 1:** Baseline patient characteristics

**Characteristic**	**Sunitinib (*****N*****=4543)**
Median age (range), years	59.0 (19.0–89.0)
Age ⩾65 years, *n* (%)	1485 (33)
Male/female, *n* (%)	3364/1179 (74/26)
**ECOG performance status,** ***n*** **(%)**^a^	
0	1868 (41)
1	1949 (43)
2	547 (12)
3	80 (2)
4	7 (<1)
**Histology,** ***n*** **(%)**^a^	
Clear cell	4010 (88)
Non-clear cell	532 (12)
Prior nephrectomy, *n* (%)^a^	4044 (89)
**Disease sites,** ***n*** **(%)**	
Lung	3469 (76)
Lymph nodes	2333 (51)
Bone	1593 (35)
Liver	1236 (27)
Brain	338 (7)
**Prior systemic therapy,** ***n*** **(%)**	
Antiangiogenic^b^	440 (10)
Cytokine	3096 (68)
**Modified risk groups based on published MSKCC data,** ***n*** **(%)**a,^c^	
Favourable	915 (20)
Intermediate	1495 (33)
Poor	1177 (26)
**Risk groups based on the IMDC prognostic model,** ***n*** **(%)**a,^d^	
Favourable	988 (22)
Intermediate	2188 (48)
Poor	889 (20)

Abbreviations: ECOG=Eastern Cooperative Oncology Group; IMDC=International Metastatic Renal-Cell Carcinoma Database Consortium; LLN=lower limit of normal; MSKCC=Memorial Sloan-Kettering Cancer Center; ULN=upper limit of normal.

a Number (%) of patients with missing data: ECOG performance status=92 (2), histology=1 (<1), prior nephrectomy =199 (4), modified risk groups based on published MSKCC data (Motzer *et al*, 2002, 2004)=956 (21), risk groups based on the IMDC prognostic model (Heng *et al*, 2009, 2013)=478 (11).

b Included sorafenib and bevacizumab.

c Risk factors were ECOG performance status ⩾2, haemoglobin <LLN, and corrected serum calcium >10 mg dl^−1^; patients without prior cytokine treatment also had lactose dehydrogenase >1.5 × ULN and time to interferon-alfa use of <1 year as risk factors (Motzer *et al*, 2002, 2004). Patients with prior cytokine therapy were assigned to favourable, intermediate, or poor risk groups if 0, 1, or >1 risk factors were present, respectively. Patients without prior cytokine treatment were assigned to favourable, intermediate, or poor risk groups if 0, 1 or 2, or >2 risk factors were present, respectively.

d Risk factors were ECOG performance status >1, time from diagnosis to study treatment <1 year, haemoglobin<LLN, calcium>ULN, neutrophil count >ULN, and platelet count > ULN (Heng *et al*, 2009, 2013). Patients with 0, 1 or 2, or >2 risk factors were assigned to the favourable, intermediate, or poor risk groups, respectively.

**Table 2:** Treatment-related adverse events of interest and those that occurred in ⩾10% of the modified intent-to-treat population (*N*=4543)

**Adverse event**	**Grade 1/2,** ***n*** **(%)**	**Grade 3/4,** ***n*** **(%)**	**Total,** ***N*** **(%)**^a^
**Non-haematologic**			
Diarrhoea	1885 (41)	237 (5)	2122 (47)
Fatigue	1406 (31)	403 (9)	1809 (40)
Nausea	1517 (33)	111 (2)	1629 (36)^b^
Decreased appetite	1295 (29)	102 (2)	1398 (31)^b^
Mucosal inflammation	1195 (26)	137 (3)	1332 (29)
Stomatitis	1144 (25)	133 (3)	1277 (28)
Vomiting	1107 (24)	143 (3)	1250 (28)
Hand–foot syndrome	909 (20)	311 (7)	1221 (27)^b^
Dysgeusia	1124 (25)	28 (1)	1152 (25)
Hypertension	837 (18)	267 (6)	1104 (24)
Asthenia	713 (16)	306 (7)	1021 (22)b,^c^
Dyspepsia	828 (18)	16 (<1)	844 (19)
Rash	734 (16)	38 (1)	772 (17)
Constipation	628 (14)	12 (<1)	641 (14)^b^
Epistaxis	585 (13)	31 (1)	616 (14)
Yellow skin	588 (13)	5 (<1)	593 (13)
Headache	495 (11)	26 (1)	521 (11)
Hypothyroidism	489 (11)	27 (1)	516 (11)
Skin discolouration	487 (11)	4 (<1)	491 (11)
Hair colour changes	481 (11)	9 (<1)	490 (11)
Dry skin	458 (10)	3 (<1)	461 (10)
Pain in extremity	413 (9)	42 (1)	455 (10)
ALT increased	94 (2)	23 (1)	117 (3)
Cardiac failure	0	13 (<1)	17 (<1)
Congestive cardiac failure	1 (<1)	13 (<1)	14 (<1)
**Haematologic**^d^			
Thrombocytopenia	741 (16)	440 (10)	1182 (26)
Neutropenia	486 (11)	315 (7)	801 (18)
Anaemia	594 (13)	203 (4)	798 (18)^b^
Leukopenia	414 (9)	97 (2)	511 (11)

Abbreviation: ALT=alanine aminotransferase.

a Eighty patients (2%) died from treatment-related adverse events (data not shown, except for cardiac failure (*n*=4) and asthenia and thrombocytopenia (both *n*=1)).

b Grade missing for one patient.

c Includes one patient with grade 5 asthenia, a 55-year-old female with a medical history of hypertension and Hodgkin's disease, who had baseline Eastern Cooperative Oncology Group performance status of 2, and massive liver metastases, and pulmonary and mediastinal metastases before the start of the study; in addition to asthenia, other treatment-related serious adverse events experienced by the patient included dyspnoea, thrombopenia, hypotension, and hypothermia.

d Related haematological adverse events with different preferred terms were collected and pooled.

**Table 3:** Tumour response according to RECIST (version 1.0) and clinical benefit

		**Prior cytokine treatment**		**Patient subgroups**				
	**All patients**^a^ (***N*****=4219)**	**Yes (*****n*****=2907)**	**No (*****n*****=1312)**	**Age ⩾65 years (*****n*****=1386)**	**Age <65 years (*****n*****=2833)**	**ECOG PS ⩾2 (*****n*****=587)**	**Non-clear cell histology (*****n*****=505)**	**Brain metastases (*****n*****=324)**
Number of evaluable patients	3353	2343	1010	1030	2323	300	379	215
Objective response, *n* (%)	660 (16)	444 (15)	216 (16)	195 (14)	465 (16)	32 (5)	42 (8)	30 (9)
Complete response, *n* (%)	63 (1)	34 (1)	29 (2)	8 (1)	55 (2)	1 (<1)	4 (1)	3 (1)
Partial response, *n* (%)	597 (14)	410 (14)	187 (14)	187 (13)	410 (14)	31 (5)	38 (8)	27 (8)
Stable disease ⩾3 months, *n* (%)	1893 (45)	1347 (46)	546 (42)	596 (43)	1297 (46)	149 (25)	217 (43)	107 (33)
Progressive disease or stable disease <3 months, *n* (%)	800 (19)	552 (19)	248 (19)	239 (17)	561 (20)	119 (20)	120 (24)	78 (24)
Clinical benefit,^b^ *n* (%)	2553 (61)	1791 (62)	762 (58)	791 (57)	1762 (62)	181 (31)	259 (51)	137 (42)

Abbreviations: ECOG PS=Eastern Cooperative Oncology Group performance status; RECIST=Response Evaluation Criteria in Solid Tumors.

a Overall, 324 patients were excluded from the modified intent-to-treat population for objective response due to non-RECIST tumour assessments. A further 866 patients were included in the analysis but were not assessed (*n*=250), not evaluable (*n*=19), or had missing data (*n*=597).

b Clinical benefit=objective response+stable disease for ⩾3 months.

**Table 4:** Summary of median progression-free survival and overall survival

		**Prior cytokine treatment**		**Patient subgroups**				
	**All patients (*****N*****=4543)**	**Yes (*****n*****=3096)**	**No (*****n*****=1447)**	**Age ⩾65 years (*****n*****=1485)**	**Age <65 years (*****n*****=3058)**	**ECOG PS ⩾2 (*****n*****=634)**	**Non-clear cell histology (*****n*****=532)**	**Brain metastases (*****n*****=338)**
Included in PFS analysis,^a^ *n*	4219	2907	1312	1386	2833	587	505	324
Median PFS (95% CI), months	9.4 (8.8–10.0)	9.3 (8.6–10.1)	9.7 (8.4–10.8)	10.1 (8.8–10.9)	9.2 (8.5–9.8)	3.5 (2.8–4.2)	6.0 (5.4–7.0)	5.3 (4.4–5.6)
Median OS (95% CI), months	18.7 (17.5–19.5)	18.4 (17.1–19.5)	19.0 (17.2–21.0)	18.1 (16.5–20.3)	18.8 (17.4–19.8)	5.7 (4.9–6.4)	12.2 (10.2–14.2)	8.2 (7.4–9.6)

Abbreviations: CI=confidence interval; ECOG PS=Eastern Cooperative Oncology Group performance status; OS=overall survival; PFS=progression-free survival; RECIST=Response Evaluation Criteria in Solid Tumors.

a Overall, 324 patients were excluded from the modified intent-to-treat population for PFS analysis because of non-RECIST tumour assessment. Survival events including all deaths were collected up to September 2008.

**Table 5:** Multivariate Cox analysis of overall survival using the IMDC prognostic factors

**Parameter**	**Parameter estimate±s.e.**	**Hazard ratio**	**95% CI**	***P*****-value**
ECOG PS >1	0.79**±**0.05	2.20	1.98–2.44	<0.0001
Time from diagnosis to treatment <1 year	0.28**±**0.04	1.32	1.21–1.44	<0.0001
Haemoglobin < LLN	0.61**±**0.05	1.84	1.68–2.01	<0.0001
Calcium>ULN	0.34**±**0.06	1.41	1.25–1.59	<0.0001
Neutrophil count > ULN	0.71**±**0.05	2.03	1.83–2.25	<0.0001
Platelet count > ULN	0.31**±**0.05	1.36	1.23–1.50	<0.0001

Abbreviations: CI=confidence interval; ECOG PS=Eastern Cooperative Oncology Group performance status; IMDC=International Metastatic Renal-Cell Carcinoma Database Consortium; LLN=lower limit of normal; s.e.=standard error; ULN=upper limit of normal.
