# Supplementary material for: Final results from the large sunitinib global expanded-access trial in metastatic renal cell carcinoma
Source: Br J Cancer. 2015 Jun 18;113(1):12–9. doi: 10.1038/bjc.2015.196 (PMC4647545; doi:10.1038/bjc.2015.196)
Supplement: Supplementary Information [file bjc2015196x1.doc]

**Supplementary Table 1.** Laboratory abnormalities identified as all-causality hepatic adverse events (*N*=4,543)

| **Adverse event** | **Grade 1,  *n* (%)** | **Grade 2,  *n* (%)** | **Grade 3,  *n* (%)** | **Grade 4,  *n* (%)** | **Grade 5, n (%)** | **Total,  *N* (%)** |
| --- | --- | --- | --- | --- | --- | --- |
| AST increased | 110 (2) | 41 (1) | 27 (1) | 4 (<1) | 0 | 182 (4) |
| ALT increased | 77 (2) | 37 (1) | 35 (1) | 3 (<1) | 0 | 152 (3) |
| ALP increased | 75 (2) | 38 (1) | 21 (<1) | 0 | 0 | 134 (3) |
| GGT increased | 23 (1) | 20 (<1) | 23 (1) | 2 (<1) | 0 | 68 (1) |
| Bilirubin increased | 28 (1) | 19 (<1) | 10 (<1) | 0 | 0 | 57 (1) |
| Abbreviation: ALP = alkaline phosphatase; ALT = alanine aminotransferase; AST = aspartate aminotransferase; GGT = Gamma-glutamyl transferase. | | | | | | |

**Supplementary Table 2.** Treatment-related non-hematologic adverse events of interest and those that occurred in ≥10% of the modified intent-to-treat population, by age (<65 [*n*=3,058] vs ≥65 years [*n*=1,485])

|  | **Grade 1/2, *n* (%)** | | **Grade 3/4, *n* (%)** | | **Total, *N* (%)^a^** | |
| --- | --- | --- | --- | --- | --- | --- |
| **Adverse event** | **Age <65 years** | **Age ≥65 years** | **Age <65 years** | **Age ≥65 years** | **Age <65 years** | **Age ≥65 years** |
| Diarrhea | 1,301 (43) | 584 (39) | 158 (5) | 79 (5) | 1,459 (48) | 663 (45) |
| Fatigue | 957 (31) | 449 (30) | 235 (8) | 168 (11) | 1,192 (39) | 617 (42) |
| Nausea | 1,048 (34) | 469 (32) | 67 (2) | 44 (3) | 1,116 (36)**^b^** | 513 (35) |
| Decreased appetite | 818 (27) | 477 (32) | 59 (2) | 43 (3) | 877 (29) | 521 (35)**^b^** |
| Mucosal inflammation | 812 (27) | 383 (26) | 77 (3) | 60 (4) | 889 (29) | 443 (30) |
| Stomatitis | 785 (26) | 359 (24) | 86 (3) | 47 (3) | 871 (28) | 406 (27) |
| Vomiting | 762 (25) | 345 (23) | 92 (3) | 51 (3) | 854 (28) | 396 (27) |
| Hand–foot syndrome | 637 (21) | 272 (18) | 229 (7) | 82 (6) | 866 (28) | 355 (24)**^b^** |
| Dysgeusia | 747 (24) | 377 (25) | 18 (1) | 10 (1) | 765 (25) | 387 (26) |
| Hypertension | 583 (19) | 254 (17) | 158 (5) | 109 (7) | 741 (24) | 363 (24) |
| Asthenia | 486 (16) | 227 (15) | 158 (5) | 148 (10) | 645 (21)**^c^** | 376 (25)**^b^** |
| Dyspepsia | 582 (19) | 246 (17) | 10 (<1) | 6 (<1) | 592 (19) | 252 (17) |
| Rash | 526 (17) | 208 (14) | 29 (1) | 9 (1) | 555 (18) | 217 (15) |
| Constipation | 397 (13) | 231 (16) | 8 (<1) | 4 (<1) | 406 (13)**^b^** | 235 (16) |
| Epistaxis | 386 (13) | 199 (13) | 21 (1) | 10 (1) | 407 (13) | 209 (14) |
| Yellow skin | 414 (14) | 174 (12) | 3 (<1) | 2 (<1) | 417 (14) | 176 (12) |
| Headache | 359 (12) | 136 (9) | 17 (1) | 9 (1) | 376 (12) | 145 (10) |
| Hypothyroidism | 328 (11) | 161 (11) | 18 (1) | 9 (1) | 346 (11) | 170 (11) |
| Skin discoloration | 356 (12) | 131 (9) | 1 (<1) | 3 (<1) | 357 (12) | 134 (9) |
| Hair color changes | 407 (13) | 74 (5) | 6 (<1) | 3 (<1) | 413 (14) | 77 (5) |
| Dry skin | 332 (11) | 126 (8) | 1 (<1) | 2 (<1) | 333 (11) | 128 (9) |
| Pain in extremity | 286 (9) | 127 (9) | 31 (1) | 11 (1) | 317 (10) | 138 (9) |
| ALT increased | 71 (2) | 23 (2) | 17 (1) | 6 (<1) | 88 (3) | 29 (2) |
| Cardiac failure | 0 | 0 | 7 (<1) | 6 (<1) | 10 (<1) | 7 (<1) |
| Congestive cardiac failure | 1 (<1) | 0 | 6 (<1) | 7 (<1) | 7 (<1) | 7 (<1) |
| Abbreviation: ALT = alanine aminotransferase.  **^a^**Eighty patients died from treatment-related adverse events (data not shown, except for cardiac failure [age <65 years, *n*=3; age ≥65 years, *n*=1] and asthenia [age <65 years, *n*=1]).  **^b^**Grade missing for 1 patient.  **^c^**Includes 1 patient with grade 5 asthenia, a 55-year-old female with a medical history of hypertension and Hodgkin’s disease, who had baseline Eastern Cooperative Oncology Group performance status of 2, and massive liver metastases, and pulmonary and mediastinal metastases before the start of the study; in addition to asthenia, other treatment-related serious adverse events experienced by the patient included dyspnea, thrombopenia, hypotension, and hypothermia. | | | | | | |

**Supplementary Table 3.** Treatment-related non-hematologic adverse events of interest and those that occurred in ≥10% of the modified intent-to-treat population, by ECOG performance status (0 or 1 [*n*=3,817] vs. ≥2 [*n*=634])

|  | **Grade 1/2, *n* (%)** | | **Grade 3/4, *n* (%)** | | **Total, *N* (%)^a^** | |
| --- | --- | --- | --- | --- | --- | --- |
| **Adverse event** | **ECOG PS  0 or 1** | **ECOG PS ≥2** | **ECOG PS  0 or 1** | **ECOG PS ≥2** | **ECOG PS  0 or 1** | **ECOG PS ≥2** |
| Diarrhea | 1,696 (44) | 151 (24) | 207 (5) | 27 (4) | 1,903 (50) | 178 (28) |
| Fatigue | 1,291 (34) | 101 (16) | 337 (9) | 58 (9) | 1,628 (43) | 159 (25) |
| Nausea | 1,340 (35) | 149 (24) | 91 (2) | 19 (3) | 1,432 (38)**^b^** | 168 (26) |
| Decreased appetite | 1,143 (30) | 126 (20) | 83 (2) | 19 (3) | 1,226 (32) | 146 (23)**^b^** |
| Mucosal inflammation | 1,028 (27) | 144 (23) | 114 (3) | 21 (3) | 1,142 (30) | 165 (26) |
| Stomatitis | 1,018 (27) | 103 (16) | 105 (3) | 23 (4) | 1,123 (29) | 126 (20) |
| Vomiting | 957 (25) | 129 (20) | 115 (3) | 24 (4) | 1,072 (28) | 153 (24) |
| Hand–foot syndrome | 828 (22) | 67 (11) | 289 (8) | 18 (3) | 1,117 (29) | 86 (14)**^b^** |
| Dysgeusia | 1,021 (27) | 83 (13) | 25 (1) | 2 (<1) | 1,046 (27) | 85 (13) |
| Hypertension | 774 (20) | 53 (8) | 248 (6) | 16 (3) | 1,022 (27) | 69 (11) |
| Asthenia | 634 (17) | 54 (9) | 251 (7) | 43 (7) | 886 (23)**^b^** | 98 (15)**^c^** |
| Dyspepsia | 763 (20) | 58 (9) | 13 (<1) | 2 (<1) | 776 (20) | 60 (9) |
| Rash | 659 (17) | 68 (11) | 36 (1) | 1 (<1) | 695 (18) | 69 (11) |
| Constipation | 555 (15) | 57 (9) | 8 (<1) | 4 (1) | 564 (15)**^b^** | 61 (10) |
| Epistaxis | 526 (14) | 51 (8) | 26 (1) | 5 (1) | 552 (14) | 56 (9) |
| Yellow skin | 510 (13) | 66 (10) | 5 (<1) | 0 | 515 (13) | 66 (10) |
| Headache | 457 (12) | 33 (5) | 19 (<1) | 7 (1) | 476 (12) | 40 (6) |
| Hypothyroidism | 454 (12) | 28 (4) | 27 (1) | 0 | 481 (13) | 28 (4) |
| Skin discoloration | 441 (12) | 42 (7) | 3 (<1) | 1 (<1) | 444 (12) | 43 (7) |
| Hair color changes | 442 (12) | 31 (5) | 9 (<1) | 0 | 451 (12) | 31 (5) |
| Dry skin | 423 (11) | 28 (4) | 3 (<1) | 0 | 426 (11) | 28 (4) |
| Pain in extremity | 378 (10) | 25 (4) | 38 (1) | 2 (<1) | 416 (11) | 27 (4) |
| ALT increased | 90 (2) | 4 (1) | 21 (1) | 2 (<1) | 111 (3) | 6 (1) |
| Cardiac failure | 0 | 0 | 10 (<1) | 2 (<1) | 14 (<1) | 2 (<1) |
| Congestive cardiac failure | 1 (<1) | 0 | 12 (<1) | 1 (<1) | 13 (<1) | 1 (<1) |
| Abbreviation: ALT = alanine aminotransferase. **^a^**Eighty patients died from treatment-related adverse events (data not shown, except for cardiac failure [ECOG PS 0 or 1, *n*=4] and asthenia [ECOG PS ≥2, *n*=1]).  **^b^**Grade missing for 1 patient.  **^c^**Includes 1 patient with grade 5 asthenia, a 55-year-old female with a medical history of hypertension and Hodgkin’s disease, who had baseline Eastern Cooperative Oncology Group performance status of 2, and massive liver metastases, and pulmonary and mediastinal metastases before the start of the study; in addition to asthenia, other treatment-related serious adverse events experienced by the patient included dyspnea, thrombopenia, hypotension, and hypothermia. | | | | | | |
